# Community knowledge, attitudes, and preferences towards vitamin A supplementation in a semi-rural health zone, Kasai Oriental, Democratic Republic of the Congo: a mixed-methods study

**DOI:** 10.1080/16549716.2026.2728856

**Published:** 2026-09-07

**Authors:** Landry Egbende, Ingunn M.S. Engebretsen, Josué Lukusa, Branly Mbunga, Paulin B. Mutombo, Mala Ali Mapatano, Pierre Z. Akilimali

**Affiliations:** a Department of Nutrition, Kinshasa School of Public Health, University of Kinshasa, Kinshasa, Democratic Republic of the Congo; b Department of Global Public Health and Primary Care, Centre for International Health, University of Bergen, Bergen, Norway; c Patrick Kayembe Research Centre, Kinshasa School of Public Health, University of Kinshasa, Kinshasa, Democratic Republic of the Congo

**Keywords:** Awareness, preferences, vitamin A supplementation, Democratic Republic of the Congo, attitudes

## Abstract

**Background:**

Vitamin A deficiency remains a critical public health challenge and affects children under 5 years of age in the Democratic Republic of the Congo.

**Objective:**

We investigated the community-level knowledge, preferences, and attitudes towards vitamin A supplementation in a semi-rural Health Zone, Kasaï-Oriental province.

**Methods:**

A sequential mixed-methods approach was applied; qualitative data included in-depth interviews with community leaders and focus-group discussions with parents (*n* = 79); quantitative data included a multi-stage sampling survey among caregivers of children under age 5 (*n* = 420). Qualitative data were analysed using thematic analysis supported by NVivo 14. The primary respondents for the survey were mothers/caregivers of children under 5-years old. A negative binomial regression analysis was used to determine the factors associated with vitamin A knowledge scores using Stata 17.

**Results:**

The majority (95.5%) of respondents had relatively low knowledge scores (≤4). As reported by mother/caregiver, knowledge was positively associated with the father’s level of education and information from community health workers (adjusted incidence rate ratio [95% confidence interval] = 1.35 [1.02–1.78] and 1.56 [1.08–2.23], respectively). The community members had a positive attitude regarding vitamin A supplementation. In addition, the administration of vitamin A in health centres and at households were equally denoted as the preferred approaches.

**Conclusion:**

Community health workers played a key role in informing the community regarding vitamin A supplementation. Strengthening the competence and interactions of community health workers with community members is a key factor for successful vitamin A supplementation delivery.

## Paper Context

- **Main findings:** Community members have low-level knowledge about vitamin A, which was based on information provided by community health workers, but they have apositive attitude and a preference for receiving vitamin A at the health centre and in their households.
- **Added knowledge:** Community health workers play an important role and can be trained to improve community knowledge regarding vitamin A supplementation and vitamin A coverage among children under 5 years of age.
- **Global health impact for policy and action:** This study highlights the need to improve community members’ knowledge of vitamin A supplementation, as awareness is an important facilitatory factor in acceptance and strengthens the abilities of community health workers.

## Background

Vitamin A deficiency (VAD) is one of the important public health issues worldwide [1–3]. As one of the four major nutritional deficiencies worldwide next to iron, iodine, and zinc deficiencies, VAD can lead to deficits in growth and development of children, loss of vision, and susceptibility to infections and their exacerbation [4,5]. Notably, VAD compromises epithelial integrity and immune function, impairing both innate and adaptative immune responses and thereby increasing children’s vulnerability to infections such as measles and diarrhoeal diseases [4,5]. According to the World Health Organization (WHO), 190 million preschool children and 19 million pregnant women are affected by VAD worldwide [3,6,7]. Despite the decline in the prevalence of VAD worldwide, possibly owing to widespread vitamin A supplementation (VAS) [8], and generally improved economies, VAD remains a major challenge, particularly in low-income countries [9]. Therefore, in these areas, routine VAS is a key strategy to reduce VAD, mortality, and morbidity in preschool children [10,11].

In the Democratic Republic of the Congo (DRC), VAS has been implemented for over two decades. However, coverage remains suboptimal, with only 53% of children supplemented, according to the 2023–2024 Democratic Health Survey (DHS III) [12]. In the Kasaï-Oriental province, child malnutrition persists [13,14], and according to the DHS III, approximately 21% of children under 5 years of age experienced diarrhoea based on a 2-week recall, and only a few of those affected sought treatment [12]. Given the well-established link between VAD and increased susceptibility to diarrhoeal disease, this diarrhoea burden highlights the urgent need to treat VAD and other deficiencies in this province.

There are several barriers that limit access to VAS at all levels of the health system – from policymakers to community members. Decisions made by policymakers must consider the views of community members to ensure greater ownership by the community. Among the community factors that limit access, knowledge remains a key factor that improves good practices [14]. Adequate knowledge about vitamin A is important; however, such knowledge in itself may not lead to higher VAS uptake. Strengthening trust in health services, improving access, and incorporating the preferences of the community members are other factors that could enhance VAS coverage [15]. Furthermore, communities may prefer other forms of intervention to those that are imposed on them, especially as most interventions are not based on recommendations from the population, and this could be a barrier to accepting these interventions [16].

The current literature on the DRC has largely focused on documenting the community-level vitamin A uptake, and thus offers limited insight into the social and behavioural dimensions that underpin VAS practices [17–21]. Critically, no study to our knowledge has systematically explored community members’ knowledge, attitudes, and preferences regarding VAS in Kasaï-Oriental – a province characterised by persistent child malnutrition and suboptimal supplementation coverage. Without understanding what communities know, believe, and prefer regarding VAS, it is difficult to design targeted communication strategies or delivery approaches that resonate with local populations. Policymakers consider the limited community-level awareness as a major barrier of VAS [22]; however, to our knowledge, no study has evaluated the community-level perspective regarding VAS. This knowledge gap could hinder the ability to tailor and adapt national VAS strategies to better align with population needs and expectations. Thus, this study used a mixed methodology to capture the community-level knowledge, preferences, and attitudes towards VAS in the Kasai Oriental province to develop tailored strategies to improve VAS uptake. This approach was necessary because the social and behavioural factors underlying VAS uptake in this context were insufficiently understood to be captured by quantitative instruments alone, and because triangulating qualitative and quantitative data strengthens the validity and actionability of the findings.

## Methods

### Study design

This study adopted a sequential exploratory mixed-methods approach where the qualitative data collection and analysis preceded the quantitative component. The qualitative data included in-depth interviews (IDIs) with community leaders and focus group discussions (FGDs) separately with the mothers and fathers of children under 5 years of age. The study was carried out between 15 July and 15 August 2024. The quantitative data collection included a survey, during 5 May–1 June 2025, with mothers as the primary respondents, or caregivers if the mothers were not present, of children under 5 years of age to assess their level of knowledge, preferences, and attitudes regarding vitamin A. Mixing was done at two levels: first, the qualitative findings informed the design of the quantitative data collection tool; second, mixing was undertaken at the analysis and interpretation stage. The qualitative findings were fully analysed prior the development of the quantitative survey instrument. This study design was chosen because it combines the exploratory strength of qualitative research with the generalisability of quantitative methods, which facilitates measurement of the knowledge of community members [23]. This method is particularly suited to informing the adaptation of VAS. This manuscript was prepared in accordance with EQUATOR Network guidelines. The qualitative component adheres to the Consolidated Criteria for Reporting Qualitative Research (COREQ) whereas the quantitative component adheres to the Strengthening the Reporting of Observational Studies in Epidemiology (STROBE). This mixed-methods study was reported in accordance with the Good Reporting of a Mixed Methods Study (GRAMMS) checklist (Supplemental table 3).

### Setting

This study was conducted in the province of Kasai-Oriental. The qualitative part of the research was carried out in five health zones – Bonzola, Bipemba, Nzaba, Miabi, and Cilenge – details of these health zones have been previously described [14]. The quantitative part concerned only one of the five health zones – the Nzaba health zone, which is among those most affected by malnutrition in the Kasai Oriental province [24], including outbreaks of measles epidemics, and benefits from several support programmes to combat malnutrition.

## Sampling approach and data collection

### Qualitative data methods

#### Sampling and study population

Participants were purposively selected from the five health zones in the province of Kasai-Oriental. A total of 10 FGDs were conducted, with two FGDs in each health zone, and one IDI was conducted with the community leader in each health zone, which added up to five IDIs (Table 1). The focus groups included eight to nine participants, and in each health zone, one FGD was conducted with fathers and one with mothers of children under 5 years of age. Participants had to have resided in the area for at least 6 months for eligibility to be included in the FDGs.

**Table 1.** Demographic characteristics of the participants in the qualitative data collection.

Characteristic	IDIs – Community Leaders (n = 5)	FGDs – Community Members (n = 74)
**Age, years**		
20–30	–	37 (50.0%)
31–40	–	37 (50.0%)
41–50	–	–
>50	5 (100.0%)	–
**Sex**		
Female	1 (20.0%)	37 (50.0%)
Male	4 (80.0%)	37 (50.0%)
**Level of Education**		
No education	–	–
Primary	–	12 (16.0%)
Secondary	2 (40.0%)	62 (84.0%)
Higher	3 (60.0%)	–

#### Data collection and management

All IDIs were conducted by the first author in French, and the FDGs were conducted by the first author, along with a translator, in the local language (Tshiluba). The IDIs and FGDs ended when saturation was reached. All IDIs and FGDs were audio recorded using digital audio recorders, and notes were taken. The audios were transcribed verbatim into French, and the FDGs were translated before being transcribed in French. The transcripts were translated into English. Transcripts were quality‑controlled by re-listening to the audio recordings and comparing them with transcripts.

### Quantitative data methods

#### Sampling and study population

The participants included mothers or caregivers of children under 5 years in the selected households. The respondent identified as the primary caregiver was selected for participation without assessing biological or legal relationship to the child. The Nzaba health zone comprises 18 health areas, and 7 health areas were excluded owing to their extremely difficult accessibility because of multiple instances of soil erosion, which hindered data collection. We used a multi-stage sampling approach in this study; 7 of the 11 accessible health areas were selected using probability proportional to size sampling. In each area, 6 avenues were chosen by simple random sampling prior to household selection. Household listing and mapping was done in the selected avenues. Ten households were systematically sampled within each avenue having at least one child aged 6 to 59 months – the target of VAS campaigns.

#### Sample size estimation

The formula for estimating a single proportion was used. Where Z was the critical value of the normal distribution (1.96), α is 0.05. P is the proportion of mothers/caregivers with good knowledge of vitamin A, which is unknown (50%) and a precision d of 0.06 was used. We adjusted with the design effect of 1.5, which is commonly used in the DRC owing to the absence of local intra-class correlation (ICC) estimates [25,26]. Therefore, the minimal sample size was estimated at 400 children; with a 5% non-response rate, the calculation yielded a total sample size of 420.

#### Data collection and management

Data collection was carried out by interviewers who were trained for 4 days in the use of tablets using the surveyCTO application (SurveyCTO | Login). The questionnaire was pre-tested to identify and address any inconsistencies. During data collection, the first author (LE) conducted regular quality control checks of the data uploaded to the server. The data included the sociodemographic characteristics of households, knowledge, preferences, and attitudes that included the age, educational level of respondents, and socioeconomic variables. Participants who did not attend school and those with primary education were grouped as having no or low education, and those with secondary and higher education were grouped as having higher education. The socioeconomic variables included questions on ownership of durable assets and whether they had income-generating activities and, if so, what they were. To assess vitamin A knowledge scores, a series of questions were included that covered the importance of vitamin A (4 items), sources of vitamin A (6 items), frequency of VAS (1 item), and the target of VAS (1 item) in the context of the DRC. The 4 items on the importance of vitamin A included vision, defence, growth and development, and reproduction [27], and 6 items for sources included green leafy vegetables, carrots and orange sweet potatoes, other orange or yellow fruits, animal liver and organs, other meats, and palm oil. The knowledge of vitamin A benefits and dietary sources was assessed using an interviewer-administered open-ended checklist. Respondents were asked to freely cite all benefits and food sources they knew about, and the interviewer recorded each mentioned item independently (yes/no per item). Each correct answer was scored as 1, and a total score was created as the sum of all scores. The attitude was measured using a Likert scale (strongly agree, agree, neutral, disagree and strongly disagree) on the extent to which VAS was beneficial for children. Preference was measured by asking the preferred method and location of receiving vitamin A. As this is an area of food insecurity, the household food security status was measured using the Household Food Insecurity Access Scale (HFIAS).

## Data analysis

### Qualitative data

Data were analysed according to the inductive thematic approach using NVivo 14 software. The transcripts were then read and reread several times to ensure familiarisation with the data. After this phase, the coding process began, and similar codes were grouped together to form preliminary themes. To increase coding validity, independent codes were created from a few interviews by two researchers and were compared until a coding framework was agreed on. Finally, triangulation of IDIs and FGDs was used to validate the data. The analyses were carried out by a team comprising both healthcare professionals and a sociologist with experience in qualitative research. In addition, the IDIs and FGDs were conducted by the first author. The main themes were discussed in the research team for robust interpretation.

### Quantitative data

All statistical analyses were performed using Stata 17 software. Descriptive statistics were used to summarize participant characteristics, attitudes, and preferences. The variables were presented as frequencies.

To assess the internal consistency of the knowledge scale, we employed both the Kuder–Richardson Formula 20 (KR-20) and Cronbach’s alpha. These methods are particularly suited for scales comprising binary items and provide a robust measure of reliability. The KR-20 specifically addresses the nature of dichotomous items and calculates the average of all possible split-half coefficients to gauge internal consistency. In addition, Cronbach’s alpha enables broader comparisons with traditional reliability practices and offers insight into inter-item correlations. Utilising both the KR-20 and Cronbach’s alpha ensures a comprehensive evaluation of the scale’s reliability. A high reliability coefficient from either measure would indicate that the items consistently capture the construct of knowledge about vitamin A; however, a lower coefficient would suggest the need for further item review. This dual approach enhances the validity of our findings and supports future revisions to the knowledge scale.

The reliability analysis for the knowledge score scale yielded a Cronbach’s alpha of 0.65. This indicates moderate internal consistency among the items. Cluster-adjusted (with health areas as the unit) negative binomial regression analyses were used to determine the factors associated with the vitamin A knowledge score. The knowledge score was not normally distributed even after logarithmic transformation. Crude and adjusted incidence rate ratios (IRRs) were reported in the multivariable analysis. The model was constructed by a forward stepwise-selection approach with covariate inclusion probability (*p* value of crude IRR) <0.20 in the bivariate analysis. Additional variables that were previously reported to influence knowledge were included in the model.

Principal component analysis (PCA) was used to generate a composite wealth index that reflected the household socioeconomic status. The analysis included key household assets and living condition variables, such as ownership of goods (e.g. radio, television, mobile phone, freezer, computer, bicycle, motorcycle, vehicle, lands, farmed or poultry) and access to electricity. The suitability of the data for PCA was assessed using the Kaiser–Meyer–Olkin (KMO) scale, and all items with poor KMO (<0.65) were removed. The first component was retained to assign a wealth score to each household, which was then used to classify households into five socioeconomic quintiles – from the poorest to the richest. A significance level of *p* < 0.05 was used.

## Integration of qualitative and quantitative data

Qualitative and quantitative data were integrated at several stages, particularly at the design and interpretation levels. At the design level, qualitative data were used to adapt the quantitative research questionnaire, which quantified the information collected during the qualitative analysis. The qualitative data enabled the construction of the essential variables for the quantitative phase. At the interpretation level, the integration enabled the qualitative data from phase 1 to be linked to the quantitative data from phase 2, presented in a joint display (Table 6). The various quotes from phase 1 were compared with the frequencies and proportions in phase 2, and these results were discussed as a whole.

## Ethics

This study adhered to the four ethical principles established in the Declaration of Helsinki, receiving ethical approval from the Ethics Committee of the Kinshasa School of Public Health (reference number: ESP/CE/67B/2024), initially granted on 28 March 2024 and subsequently updated on 5 May 2025 and the Norwegian Institutional Review Board (REK West; reference number: 645,696). Autonomy was maintained as participants provided written informed consent following a comprehensive elucidation of the study’s objectives. Participants were apprised that their participation was voluntary and that they could disengage from the talks at any moment. Confidentiality was ensured by excluding all identifying information – both from transcripts and the questionnaire – which guaranteed participant confidentiality. Thus, all citations were marked according to a site letter (A, B, C, D), not by geographical location.

## Results

This study included 10 FGDs with 8–9 participants (*n* = 74) and 5 IDIs with community leaders (CLs), with one CL in each health zone (Table 1). For the quantitative part, a total of 420 participants responded to our questionnaire, and their sociodemographic characteristics are described below (Table 2). All the respondents were mothers or female caregivers. Furthermore, the population generally had low education, lived in populated households, and experienced high food insecurity. The qualitative and quantitative results are presented together, following the themes below: First, knowledge is presented, including knowledge of vitamin A benefits, sources, supplementation activities, and the determinants of adequate knowledge. Thereafter, attitudes and preferences regarding VAS-related activities are presented.

**Table 2.** Sociodemographic characteristics of the survey respondents.

Variables	n = 420	%
**Age of mother/caregiver, years**		
<20	22	5.3
20–34	237	56.4
≥35	161	38.3
**Level of education of mother/caregiver**		
Low	400	95.2
High	20	4.8
**Level of education of the father**		
Low	298	70.9
High	122	29.1
**Household size**		
≤6	150	35.7
≥7	270	64.3
**Income generating activities**		
No	15	3.6
Yes	405	96.4
**Level of food security**		
Food secure	9	2.1
Middle food insecurity	17	4.1
Moderate food insecurity	40	9.5
Severe food insecurity	354	84.3
**Quintile of SES**		
Lowest	84	20.0
Lower	84	20.0
Middle	84	20.0
Higher	88	21.0
Highest	80	19.0

Note: **SES** = socioeconomic status.

## Knowledge of vitamin A benefits, sources, and supplementation activities

The benefits of vitamin A, particularly in vision and strengthening the immune system, were recognised by the majority of respondents in the FGDs and IDIs. They emphasised the importance of vitamin A in preventing the occurrence of certain epidemics, such as measles. They said:

According to the teachings we received from healthcare workers who come to educate us, Vitamin A is given to children from 6 to 59 months and helps with vision and growth. It also protects against diseases like poor distant vision and aids in the child’s growth. (P6, FGD, A, male)

This vitamin helps with vision, overall health, and protects against outbreaks like polio and measles. A child who has taken vitamin A is protected from these diseases, especially concerning vision. (P4, FGD, B, female)

Vitamin A was perceived by a few respondents as an unknown nutrient. The role of this nutrient, including the importance of organised supplementation campaigns, was not recognised. Some respondents said:

Often, those who come with Vitamin A do not explain to us that it is a particular vaccine or the importance of each vaccine. If you listen carefully to what another father says, you will notice that he does not have enough information about Vitamin A. That’s why we ask for more awareness efforts. (P1, FGD, C, male)

Personally, I didn’t know what Vitamin A was until I heard from other mothers. I only knew that vaccines protect children from minor illnesses. (P6, FGD, B, female)

Awareness-increasing activities on the importance of vitamin A are generally carried out before the campaign by the CHWs, and the majority of respondents considered them the main source of information on vitamin A:

Information about Vitamin A comes from community workers using megaphones to announce vaccination sessions at health centers. I also receive information from health personnel. I find this approach effective because it helps avoid some diseases. I have never sought VAS outside of what my child receives at home. (P5, FGD, A, female)

We receive information from community workers and health personnel who visit households, which helps us bring our children for VAS (P3, FGD, A, female)

In addition, information on vitamin A was also disseminated through community radio stations.

We receive a lot of information from the radio because many of us do not live close to the center. (P3, FGD, C, male)

Findings from the survey indicated that half of the respondents had no knowledge, as mentioned on the knowledge scale, about vitamin A (Supplemental table 1). The highest score was 7/12, and 95% had a score ≤ 4. Additionally, 38.5% mentioned infection prevention; and 19.0% recognised good vision as benefits of vitamin A. Among the sources of vitamin A, green leafy vegetables and palm oil were cited by 31.2% and 28.6% as important sources, respectively. The majority of respondents were unaware of the target group and frequency of VAS (Table 3).

**Table 3.** Knowledge of vitamin A benefits, sources, and VAS.

Variables	n	%
**Heard about vitamin A**		
No	135	32.1
Yes	285	67.9
**Source of information about vitamin A**		
Community health workers (CHWs)	129	45.3
Other sources (besides CHWs)	156	54.7
**Health benefits for vitamin A**		
Prevents infections/diseases	82	38.5
Growth and development	64	29.9
Good vision	40	19.0
Reproduction	5	2.6
**Identified sources of Vitamin A**		
Leafy green vegetables	70	31.2
Palm oil	64	28.6
Ripe fruits with orange or yellow flesh	51	23.5
Other meat	20	10.0
Carrot, orange, or yellow-fleshed sweet potato	19	9.0
Liver, kidney, heart, or other offal	8	4.0
**Knowledge about VAS target age**		
<6 months	13	4.6
6–59 months	76	26.7
≥59 months	9	3.1
Don’t know	187	65.6
**Knowledge about VAS frequency**		
Once a year	40	14.0
Every month	10	3.5
Every 6 months	54	18.9
On demand	5	1.8
Don’t know	176	61.8

Overall, the majority of respondents had a vitamin A knowledge score of ≤4 (Supplemental Table 1). Using a negative binomial regression model, the analysis revealed that a father’s higher level of education improved the vitamin A knowledge score, with an incidence rate ratio (IRR) of 1.35 (95% CI: 1.02–1.78). Additionally, households that received visits from CHWs had a higher knowledge score, with an adjusted incidence rate ratio (aIRR) of 1.56 (95% CI: 1.08–2.23; Table 4).

**Table 4.** Factors associated with vitamin A knowledge scores using negative binomial regression.

	Unadjusted			Adjusted		
Variables	IRR	95% CI	*p* value	IRR	95% CI	*p* value
**Age of mother/caregiver, years**						
<20 years	1					
20–34	1.20	(0.62–2.34)	0.586	1.26	(0.58–2.70)	0.557
≥35	1.59	(0.81–3.11)	0.175	1.45	(0.62–3.39)	0.394
**Level of education of the mother/caregiver**						
Low	1					
High	1.25	(0.93–1.68)	0.132	1.17	(0.77–1.77)	0.462
**Level of education of the father**						
Low	1					
High	1.44	(1.06–1.96)	0.019	1.35	(1.02–1.78)	0.035
**Income generating activities**						
No	1					
Yes	1.09	(0.74–1.62)	0.646	1.19	(0.83–1.70)	0.337
**Source of information about vitamin A**						
Sources other than CHWs	1					
CHWs	1.59	(1.11–2.28)	0.011	1.56	(1.08–2.23)	0.016

Note: **aIRR**: adjusted incidence rate ratio. aIRR was adjusted for the following variables: age of mother/caregiver, level of education of the mother/caregiver, level of education of the father, income generating activities and source of information about vitamin A; CHW: community health workers; **IRR**: incidence rate ratio.

## Attitudes and preferences regarding VAS-related activities

In general, our respondents had a favourable attitude towards VAS given the advantages of this intervention, as several acknowledged.

I find VAS sufficient, and I am satisfied because it helps children maintain good health even when they have a fever, the child does not become too weak. (P2, FGD, C, female)

VAS is satisfactory for me because before sending anything to the community, it has been tested and proven to be suitable for our children. I can only say that it is sufficient because only healthcare personnel know what to do. (P8, FGD, C, female)

In addition, regarding VAS preferences, some respondents in the IDIs and FGDs would like to receive vitamin A routinely at health centres, while others preferred campaigns with multiple visits.

I recommend adopting a regular schedule for vitamin A supplementation, similar to the routine vaccinations. Organizing regular vitamin, A sessions, like for vaccines, could improve coverage. It’s also important to have specific campaigns dedicated solely to vitamin A to make people understand its importance, rather than just associating it with vaccinations. (CL, D)

I think there should be more sessions for vitamin A administration because some children missed the previous distribution, and you can see them with sore and burning eyes. This suggests that increasing the frequency of administration would ensure that no child misses out. As the population grows, vitamins can run out, and more frequent organization would help ensure everyone is covered. (P7, FGD, B, female)

Overall, 48.1% of respondents strongly agreed that VAS is beneficial for children in the survey. Regarding preferences for the method of receiving vitamin A, VAS was mentioned by approximately 43.2%, and approximately 21.4% would prefer to receive vitamin A through natural foods. Furthermore, the preferred venue for receiving vitamin A was the health centre and at home, with equivalent proportions (Table 5).

**Table 5.** Participants’ attitudes and preferences regarding VAS.

Variables	n = 285	%
**Attitudes** **I consider VAS to be beneficial for children**		
Strongly agree	137	48.1
Agree	115	40.4
Neutral	29	10.2
Disagree	2	0.7
Strongly disagree	2	0.7
**Preferences** **Preferred method of receiving vitamin A**		
Drops/capsules (supplements)	123	43.2
Fortified foods (e.g. enriched oil or flour)	71	24.9
Natural foods (e.g. mango, liver)	61	21.4
I don’t know	30	10.5
**Preferred place for receiving VAS**		
Health centre	138	48.4
At home	138	48.4
During awareness campaigns	8	2.8
At school	1	0.4

## Integrated interpretation of qualitative and quantitative findings

The qualitative insights were indeed reinforced by the quantitative data, as illustrated in the joint display table. The convergence and interpretation of the links between qualitative and quantitative data are described in Table 6.

**Table 6.** Joint display table.

Theme	Quote	Quantitative Variables	Quantitative results	Integrated interpretation
Knowledge of vitamin A benefits, sources, and supplementation activities	*“Often, those who come with Vitamin A do not explain to us that it is a particular vaccine or the importance of each vaccine. If you listen carefully to what another father says, you will notice that he does not have enough information about Vitamin A. That’s why we ask for more awareness efforts”*	Knowledge scores	95% ≤4/12	Lack of information →Low knowledge scores
Sources of information on vitamin A	*“We receive information from community workers and health personnel who visit households, which helps us bring our children for VAS”*	Source of VAS information	Info CHWs ↑ Knowledge scores s(a|IRR ≈1.6)	CHWs→ increase knowledge regarding vitamin A
Attitudes towards vitamin A	*“I find VAS sufficient, and I am satisfied because it helps children maintain good health even when they have a fever, the child does not become too weak”*	Attitude about VAS	48% strongly agree VAS is beneficial for their children	Community members have positive attitude towards VAS→ increased acceptance
Preferences of VAS	*“I recommend adopting a regular schedule for vitamin A supplementation, similar to the routine vaccinations. Organizing regular vitamin, A sessions, like for vaccines, could improve coverage. It’s also important to have specific campaigns dedicated solely to vitamin A to make people understand its importance, rather than just associating it with vaccinations”*	Preference of VAS	≈50% domicile / ≈ 50% centre	Combination approaches where the service can be provided at the health centre with the possibility of catching up with children who have been missed for VAS in the community

## Discussion

This sequential mixed-methods study explored the community knowledge, attitudes, and preferences of the community members in the Kasai-Oriental region regarding vitamin A and quantified the key determinants of these perceptions. The qualitative data revealed limited knowledge about vitamin A among community members, and this highlighted the important role of CHWs as the primary source of information on vitamin A. These findings were confirmed by quantitative data, showing that the majority of caregivers had low knowledge about vitamin A. The results of the negative binomial regression analysis indicate that factors such as the father’s education level and the visits from community health workers significantly influence the knowledge score. Taken together, the results showed that the community members had a positive attitude regarding vitamin A. In addition, the administration of vitamin A in health centres and at households were equally denoted as the preferred approaches.

The low knowledge about vitamin A that we found in our study is consistent with the findings reported by other researchers. In Uganda, a study conducted on predictors of vitamin A-rich food consumption found that mothers did not have a good understanding of vitamin A, which would increase the risk of vitamin A deficiency in their children [28]. Low-level knowledge about vitamin A is a major determinant of VAS acceptance, as has been demonstrated by several authors and suggests that awareness campaigns are needed to fill this gap [29]. Our results showed that high knowledge about vitamin A was associated with the high educational level of the fathers. This may be explained by the influential role that fathers play in household health decision-making in sub-Saharan African contexts, where paternal authority often shapes family health behaviours [30–33]. Higher educational attainment may enhance fathers’ ability to seek, process, and act on health information – an effect that is particularly pronounced in remote areas where access to formal health services and information channels remains limited [34]. On the other hand, secondary data analysis of a dataset of children treated for diarrhoea in Bangladesh found that parents who had not attended formal school were less compliant with VAS [35]. Low-level vitamin A knowledge suggests significant gaps in health literacy in the population. These could hinder individuals’ ability to make informed health decisions, understand health risks, and utilise healthcare services effectively [36]. Furthermore, low levels of knowledge may reflect the existence of barriers to accurate information, including beliefs and misinformation within the community [14]. It is important to implement the necessary measures to disseminate accurate information using various communication channels, such as community leaders. These findings have implications in patriarchal household structures across many low- and middle-income countries (LMICs), improving father’s health literacy through targeted education programmes may be a critical and underutilised level for improving child nutrition outcomes at the population level while strengthening women’s education and participation in decision making [34].

In addition, CHWs as the main source of information on vitamin A were associated with high vitamin A knowledge scores. This finding was confirmed in the qualitative data, where CHWs were found to be fully involved in community mobilisation, and disseminated information about the vitamin A campaign through megaphones. CHWs play an important role in promoting health interventions in the community. They are involved in various awareness campaigns and help facilitate community adherence to health interventions, including VAS. The important role of CHWs has been highlighted in several studies, particularly how their level of knowledge could influence the population-level knowledge and constitute a barrier to VAS [37,38]. Strengthening the CHWs’ capacities would improve the population’s level of knowledge through the dissemination of accurate information. This finding resonates with global evidence on the central role of CHWs in last-mile health delivery, particularly in fragile systems across sub-Saharan Africa and Asia, where CHWs often represent the primary point of contact between communities and the formal health system [39]. Investments in CHW training and supervision status are therefore a globally relevant strategy for improving micronutrient supplementation coverage, as evidenced by programmes in other LMICs [40].

Community members expressed a favourable attitude towards VAS and recognised it as a crucial intervention for improving their children’s health. This positive disposition reflects broad community acceptance of the programme, and may be attributed to the nature of the intervention itself – its ease of administration, oral route of delivery, and notably favorable safetyprofile, including the absence of significant adverse effects commonly associated with other preventive interventions [14]. Similar findings have been reported in other contexts, where VAS is viewed as a trusted and beneficial public health intervention [15]. It is logical that the attitude alone does not guarantee vitamin A uptake; accordingly, several other contextual factors, such as access to services and availability of inputs, must be addressed to improve vitamin A coverage [41].

In addition, community members expressed roughly equal preference for receiving vitamin A either at health centres or through household visits. These results reflect the community’s preference for approaches that enable their children to receive vitamin A. The health centre is an opportunity that can also facilitate access to other health services [42]. However, the preference for the household visit approach is an approach that limits household expenditure and makes it easier for their children to receive vitamin A at home [43]. This leads us to propose combined approaches where the service can be provided at the health centre with the possibility of catching up with children who have been missed in the community, especially at a time when the trend towards routine vitamin A supplementation is being promoted [44]. In some countries, such as Burkina Faso, the implementation of routine community-based VAS has been perceived as a good approach and has maintained VAS coverage in line with WHO standards [45].

This mixed-methods approach provided complementary insights. The qualitative findings helped explain why knowledge scores were low in the quantitative results. Caregivers relied on CHWs for information regarding vitamin A; however, the quantitative findings showed that high knowledge scores were associated with having CHWs as a source of information about VAS. Community members expressed a preference for receiving vitamin A both at the health centre and at home. The integration of qualitative and quantitative findings demonstrates the significant problems that exist in VAS. Community members are receptive and willing to accept VAS for their children, whereas the problem is rather the approach used to deliver VAS to the community and limited access to accurate information [15]. It is important to ensure that CHWs are strengthened, as their level of knowledge determines the community’s knowledge and also facilitates community engagement. In addition, combined approaches involving supplementation at health centres and at home would improve VAS coverage [39,44]. Beyond the DRC, the application of sequential mixed-methods designs in VAS research remains limited, and our study demonstrates the value of this approach for generating actionable, context-grounded insights that can inform programme design in similar settings globally.

Though this mixed method provides important information on the level of knowledge about vitamin A and the community’s preferences regarding the desired approach to receiving vitamin A, some limitations exist. The cross-sectional design of the study does not allow for an inference of causality [46]. Additionally, the approximately nine-month interval between the qualitative and quantitative data collection phases was inherent to the sequential mixed-methods design. Although this interval was methodologically necessary, contextual factors – including seasonal variation in health-seeking behaviour or shifts in supply availability – may have introduced some inconsistency in participants’ experiences across the two phases. However, given the relatively stable health system context in the study area during this period, we consider this influence to be limited. With respect to generalizability, the quantitative survey was conducted in a single, purposively selected health zone. It limits the statistical generalizability of our findings to other health zones or provinces with different socioeconomic, geographic, or health system profiles. Our findings should therefore be interpreted as context-specific, though they may offer transferable insights for similar LMIC scaled. Furthermore, seven health areas were excluded from the quantitative survey due to physical inaccessibility, introducing a potential selection bias. However, the socioeconomic and demographic profiles of the excluded areas are broadly similar to those surveyed; this exclusion should be considered when interpreting results and extending recommendations to inaccessible areas. The scales measuring knowledge and attitudes have not been standardised or validated. The reliability analysis of the knowledge measurement scale yielded a Cronbach’s alpha coefficient of 0.65, which indicated moderate internal consistency. Moreover, attitudes were assessed using a single Likert-scale item, which may not adequately capture the full complexity of caregivers’ attitudinal dispositions toward VAS; a multi-item attitude scale would provide greater construct validity. To improve the reliability of the scale, future research should consider revising the items, conducting pilot tests, and performing factor analysis to better understand the interrelationships between items [47]. Finally, a further limitation relates to the potential for conceptual conflation between vaccines and supplements among survey respondents, a challenge commonly reported in community-based health research in LMICs where VAS is frequently co-administered with vaccination activities [14,15]. To minimise this risk, visual aids including photographs of VAS capsules were incorporated into the data collection tool to help respondents clearly distinguish VAS from vaccines.

## Conclusion

Our study highlights a critical gap in caregivers’ knowledge of vitamin A and supplementation in a resource-limited setting in the DRC. These findings carry important practical and policy implications. Strengthening the role of CHWs as trusted information channels and leveraging paternal education through targeted male engagement strategies could meaningfully improve household knowledge and VAS uptake. Culturally tailored communication campaigns, co-designed with CHWs and adapted to local contexts, represent a promising and scalable approach to bridging knowledge gaps. However, as the quantitative component of this study was conducted in a single health zone, caution is warranted in generalising these findings more broadly. Validation in diverse geographic and socio-cultural settings across the DRC and other LMICs is recommended for further research.

## Supplementary Material

Supplemental online material.docx

GRAMMS_checklist_Egbende.docx

## Data Availability

The data that support the findings of this study are available at 10.5281/zenodo.17751651.
