# Supplementary material for: Community knowledge, attitudes, and preferences towards vitamin A supplementation in a semi-rural health zone, Kasai Oriental, Democratic Republic of the Congo: a mixed-methods study
Source: Glob Health Action. 2026 Sep 7;19(1):2728856. doi: 10.1080/16549716.2026.2728856 (PMC13552312; doi:10.1080/16549716.2026.2728856)
Supplement: Supplementary file 1 [file ZGHA_A_2728856_SM5033.docx]

**Supplemental table 3**. Good Reporting of a Mixed Methods Study (GRAMMS)

| **Checklist items** | **Status** | **References** |
| --- | --- | --- |
| Justification of using mixed methods | Completed | Line 88-91: This study design was chosen because it combines the exploratory strength of qualitative research with the generalisability of quantitative methods, which facilitates measurement of the knowledge of community members. This method is particularly suited to informing the adaptation of VAS |
| Design: purpose, sequence, and priority | Completed | Line 80-86: This study adopted a sequential exploratory mixed-methods approach where the qualitative data collection and analysis preceded the quantitative component. The qualitative data included in-depth interviews (IDIs) with community leaders and focus group discussions (FGDs) separately with the mothers and fathers of children under 5 years of age. The study was carried out between 15 July and 15 August 2024. The quantitative data collection included a survey, during 5 May–1 June 2025, with mothers as the primary respondents, or caregivers if the mothers were not present, of children under 5 years of age to assess their level of knowledge, preferences, and attitudes regarding vitamin A |
| Each method: sample, collection, and analysis | Completed | Line 128-208:  **Qualitative**: 5 IDIs with community leaders, 10 FGDs with mothers and fathers, thematic analysis using NVivo 14  **Quantitative**: survey of 420 caregivers using multi-stage sampling, knowledge scale (0-12), negative binomial regression used to analyse determinants of knowledge, Stata 17 used for data analysis. |
| Integration: where, how, and who | Completed | Line 86-88: Mixing was done at two levels: first, the qualitative findings informed the design of the quantitative data collection tool; second, mixing was undertaken at the analysis and interpretation stage (joint table: line 326) |
| Limitations: one method limits another | Completed | Line 395-400 |
| Insights from mixing | Completed | Line 381-392: This mixed-methods approach provided complementary insights. The qualitative findings helped explain why knowledge scores were low in the quantitative results. Caregivers relied on CHWs for information regarding vitamin A; however, the quantitative findings showed that high knowledge scores were associated with having CHWs as a source of information about VAS. Community members expressed a preference for receiving vitamin A both at the health centre and at home. The integration of qualitative and quantitative findings demonstrates the significant problems that exist in VAS. Community members are receptive and willing to accept VAS for their children, whereas the problem is rather the approach used to deliver VAS to the community and limited access to accurate information. It is important to ensure that CHWs are strengthened, as their level of knowledge determines the community's knowledge and also facilitates community engagement. In addition, combined approaches involving supplementation at health centres and at home would improve VAS coverage. |
